# Identification of the chain-dispersing peptidoglycan hydrolase LytB of *Streptococcus gordonii*

**DOI:** 10.1371/journal.pone.0176117

**Published:** 2017-04-17

**Authors:** Riccardo Arrigucci, Gianni Pozzi

**Affiliations:** 1 Public Health Research Institute, Rutgers, The State University of New Jersey, Newark, NJ, United States of America; 2 LAMMB, Department of Medical Biotechnologies, University of Siena, Siena, Italy; Oregon Health & Science University, UNITED STATES

## Abstract

Bacterial cell division ends with the separation of the daughter cells, a process that requires peptidoglycan hydrolases (PGHs). Bacteria lacking cell separating PGHs are impaired in cell separation with the formation of long chains or clusters. We identified a gene in *Streptococcus gordonii* encoding for a putative glucosaminidase (*lytB*). The *lytB* isogenic mutant grew in long bacterial chains and resulted in impaired biofilm formation. Purified recombinant LytB showed a murolytic activity on *Micrococcus lysodeikticus* cell suspension and was able to disperse the long chains of the mutant, restoring the wild type diplococci/short chain phenotype. LytB protein was localized only in culture supernatant cell fraction of *S*. *gordonii*, and co-cultures of wild type and *lytB* mutant showed a significant reduction of bacterial chain length, indicating that LytB is a secreted enzyme. Our results demonstrate that LytB is a secreted peptidoglycan hydrolase required for *S*. *gordonii* cell separation.

## Introduction

Bacteria produce enzymes able to cleave their own protective peptidoglycan cell wall. The number of peptidoglycan hydrolases (PGHs) encoded by a bacterial genome varies among species. Gram positive bacteria from *Streptococcus* and *Lactobacillus* genus possess a relative small number of PGHs [1], while 35 peptidoglycan hydrolases have been identified in *Bacillus subtilis* [2]. PGHs are involved in many bacterial physiological processes as autolysis, competence development, biofilm formation, sporulation, cell wall elongation and turnover, cell division and cell separation, and their classification is based on the catalytic activity on the different peptidoglycan bonds: *N*-acetylmuramyl-L-Alanine amidases, endopeptidases, carboxypeptidases, *N*-acetyl-β-D-muramidases, *N*-acetyl-β-D-glucosaminidases, and lytic transglycosylases [3, 4]. PGHs have a modular structure composed by one or two catalytic domains, and a single or multiple binding domains specific for the peptidoglycan or peptidoglycan anchored surface polymers, which localize the catalytic domain of the enzyme on the substrate [1].

Several PGHs involved in daughter cells separation have been identified. In the human pathogen *Streptococcus pneumoniae*, the deletion of *lytB* gene causes the formation of long chains of non-separated cells that can be dispersed by the addition of purified recombinant LytB enzyme [5]. The same defects in cell separation were observed in *Streptococcus thermophilus cse* deletion mutant and in rod-shaped *Bacillus anthracis bslO* deletion mutant [6, 7]. Defects in cell separation following cell division were also reported in several autolysin deficient mutants of *Lactococcus lactis* (*acmA*) [8], *Listeria monocytogenes* (*murA*) [9], *Clostridium perfringens* (*acp*) [10], *Lactobacillus plantarum* (*Acm2*) [11], and *L*. *lactis* AcmD [12], indicating that cell separation requires the involvement of PGHs.

Streptococci are elongated ellipsoids cocci that divide in successive parallel planes perpendicular to their long axis. The result of this type of cell division is the formation of single cells, diplococci or small chains [13]. During streptococcal cell division a complete septum is synthesized before cell separation and then cleaved by dedicated PGHs to allow the separation of daughter cells [14]. *Streptococcus gordonii* is an oral commensal gram positve, α-hemolytic, aerotolerant coccus that grows in short chains [15]. It is a member of the *Mitis* group, which comprises other oral streptococci as *S*. *mitis*, *S*. *oralis*, *S*. *sanguis*, *S*. *parasanguis* and the human pathogen *S*. *pneumoniae* [16]. *S*. *gordonii* is involved in the primary colonization of the saliva coated tooth surfaces where, by co-aggregating with other bacterial species, it contributes to the formation of the multispecies biofilm on healthy dental surfaces [17, 18]. *S*. *gordonii* has been used as mucosal vaccine vector for heterologous gene expression of surface recombinant antigens [19–21] and, despite belonging to the GRAS (generally regarded as safe) organisms, it is also an important etiologic agent of infective endocarditis [22, 23].

Here we report the identification of LytB as a PGH required for the cell separation of *S*. *gordonii*.

## Materials and methods

### Bacterial strains and growth conditions

*S*. *gordonii* V288 (Challis) [24] and the isogenic *lytB* mutant GP1485 were grown statically in Tryptic Soy Broth (TSB, Beckton Dickinson, Italy) at 37°C in sealed tubes. Growth was monitored spectrophotometrically at 590 nm with a Spectronic 20D+ spectrophotometer (Thermo Scientific, Waltham, MA, USA). Bacterial stocks were prepared from logarithmic growth phase cultures and stored frozen at -80°C in TSB containing 10% of glycerol. *Escherichia coli* DH5α and BL21-Codon Plus (DE3) strains (Stratagene, La Jolla, CA) were cultured in Luria-Bertani medium (LB) at 37°C in agitation or in Luria-Bertani agar (LBA).

### Amino acid sequence analysis

LytB amino acid sequence was analyzed with Pfam tool available at the Wellcome Trust Sanger Institute (http://pfam.sanger.ac.uk/). Presence of signal peptides was predicted by using SignalP 4.0 Server [25], and Blast software (http://blast.ncbi.nlm.nih.gov/Blast.cgi) was used to conduct homology searches.

### Construction of an isogenic mutant

To construct the *S*. *gordonii* isogenic mutant, the *SGO_1138* gene (Gene ID: 25052579), which we named *lytB*, was inactivated by gene SOEing, essentially as previously described [26]. A 697 bp deletion from nucleotide 1,185,670 to 1,186,367 was generated using a spectinomycin resistance cassette (894bp, GenBank acc. no. AY334020) flanked by segments of *lytB* upstream and downstream genes, *SGO_1137* (698 bp, Gene ID: 25052608) and *engB* (486 bp, Gene ID: 25052554), respectively. The DNA fragment containing the antibiotic-resistance cassette flanked by complementary regions upstream and downstream the *lytB* gene was obtained by subsequent PCR reactions using the following primers: IF622 (5’-TTTGCTGAGTGCGGCTAATA-3’), IF623 (5’-AATTGCTGCTCCAACTAGAT-3’), IF100 (5’-GCTCTAGAACTAGTGGATC-3’), IF101 (5’-TTCCCTTCAAGAGCGATAC-3’) IF624 (5’-GATCCACTAGTTCTAGAGCAGTCATAACTCCTAAAAAATACTAC-3’) and IF625 (5’-GTATCGCTCTTGAAGGGAAAGTAAGAAAAGAGTTAGGAAAAGA-3’). The obtained PCR product was used to transform *S*. *gordonii* V288 competent cells. Transformants were selected for acquisition of antibiotic resistance-marker by multilayer plating procedure [26]. The resulting mutant GP1485 was confirmed by PCR and DNA sequence analysis.

### Cloning, expression and purification of recombinant LytB

The nucleotide sequence of *lytB* was amplified by PCR using *S*. *gordonii* V288 genomic DNA as template [24] using primers IF687 (5´-GGATCCAGCTAACGAAGAACAAACA-3´) and IF675 (5´-AAGCTTACGGTTCGCCCAAGCGACG-3´) (underlined sequence indicates respectively *Bam*HI and *Hind*III restriction sites). PCR products were cloned into the pGEM T-Easy vector (Promega, Madison, WI, USA), and transformed in *E*. *coli* DH5α. Transformants were selected on LBA plates containing 100 μg ampicillin ml^-1^. Recombinant plasmids were purified and digested with *Bam*HI and *Hind*III restriction enzymes. Purified DNA fragments were inserted in the expression vector pET-21b (Novagen, Madison, WI, USA). The recombinant expression plasmid pSMB630::*lytB* was transformed in *E*. *coli* BL21-CodonPlus (DE3)-RIL strain (Stratagene La Jolla, CA, USA). The resulting strain GP1905 was grown in 500 mL of LB broth containing 50 μg ampicillin ml^-1^ and 10 μg chloramphenicol ml^-1^ at 37°C with agitation at 140 rpm to an OD_600_ of 0.6, and then induced with 1mM isopropyl β-D-1-thiogalactopyranoside (IPTG) (Sigma-Aldrich, St Louis, MO, USA) for 5 h. Cells were collected by centrifugation, resuspended in Lysis Buffer (50 mM NaH_2_PO_4_, 300 mM NaCl, 10 mM imidazole, pH 8), and incubated with 0.3 mg ml^-1^ lysozyme (Sigma-Aldrich, St Louis, MO, USA) and 0.003 μg ml^-1^ DNase I (Sigma-Aldrich, St Louis, MO, USA) at 4°C for 1 h. Bacteria were lysed by three cycle of sonication, and centrifuged at 13.000 × *g* at 4°C for 30 min. The supernatant was filtered with a 0.22 μm filter (Millipore, Billerica, MA, USA), and loaded onto a Ni-NTA column (Qiagen, Hilden, Germany), according to manufacturer’s instructions. The eluate was desalted and concentrated in Tris-HCl 50 mM pH 7.5 with 10% (v/v) glycerol using a 200 ml stirred cell ultrafiltration device having a 10 KDa Amicon membrane (Millipore, Billerica, MA, USA). Proteins were quantitated with Bradford reagent (Sigma-Aldrich, St Louis, MO, USA) following manufacturer’s protocol, and stored at -20°C. The purified recombinant LytB (recLytB) was analyzed in a mini cell electrophoresis apparatus in MES buffer using a NuPAGE 4–12% Bis-Tris acrylamide gel (Invitrogen, Carlsbad, CA, USA). Protein bands were visualized by Coomassie Brillant Blue R-250 staining (Bio-Rad, Bio-Rad, Hercules, CA, USA). For Western blot analysis, proteins were transferred on a nitrocellulose membrane with the I-Blot apparatus (Invitrogen, Carlsbad, CA, USA) according to manufacturer instructions. Membrane was blocked for 1 h with 3% (w/v) BSA in TBS buffer (100 mM Tris-HCl pH 7.5, 150 mM NaCl) and incubated with Anti-Penta His primary antibody (Qiagen, Hilden, Germany) diluted 1:1,000 in TBS containing 3% (w/v) BSA for 1.5 h. Membrane was washed three times in TBS-Tween 0.05% (v/v), incubated with anti-mouse secondary antibody conjugated with alkaline phosphatase (AP) diluted 1:10,000 in TBS 3% (w/v) skim milk powder for 1.5 h, washed, and incubated with NBT-BCIP solution (Sigma-Aldrich, St Louis, MO, USA). The reaction was stopped by using dH_2_O when protein bands were clearly visible.

### Ethics statement

Animal experimentation in Italy is regulated by Decreto Legislativo 116/92 and Directive 210/63/EU. The animal protocol was approved by the “Comitato Etico Locale” of the Azienda Universitaria Ospedaliera Senese and received thereafter the relative project licence issued by the Italian Ministry of Health (193/2008-B).

### Anti-LytB antibody production

Three six-week-old female BALB/c mice (Charles River, Italy) were immunized subcutaneously with 50 μg of recLytB adsorbed to aluminum hydroxide. To obtain the aluminum-adsorbed antigen, recLytB (500 μg) was resuspended in 1 ml of 0.15 M NaCl and added to a solution of 2 mg ml^-1^ Aluminum hydroxide gel (Sigma-Aldrich, St Louis, MO, USA) in NaCl 0.15 M. The suspension was incubated in agitation at room temperature for 1 hour, and then centrifuged at 3,000 × *g*, at room temperature, for 5 min. Supernatant was collected and analyzed for unabsorbed protein with Bradford reagent. The pellet containing the adsorbed protein to aluminum gel (Al-LytB) was resuspendend in NaCl 0.15 M, and prepared for subcutaneous inoculation. An Al-LytB dose of 50 μg/mouse was delivered at day 0 and day 21. Blood samples were collected from temporal plexus at day 0 and day 35, and at day 37 by cardiac puncture. Total anti-LytB IgG serum was analyzed by enzyme-linked immunosorbent assay (ELISA) as previously described [20]. Sera were stored at -35°C.

### Lytic assay

The lytic activity of recLytB was tested on a *M*. *lysodeikticus* cell suspension. Autoclaved *M*. *lysodeikticus* ATCC 4697 cells (Sigma-Aldrich, St Louis, MO, USA) were resuspended in 50 mM Tris-HCl (pH 7.0) to an OD_450_ of 0.5 in 16-mm glass tubes. Ten-fold dilutions of the enzyme, ranging from 0.1 to 0.0001 mg ml^-1^, were added to the tubes, and mixtures were incubated at 37°C for 2 h. A cell suspension without enzyme was used as a negative control. The OD_450_ values were recorded by using a Spectronic 20D+ spectrophotometer (Thermo Scientific, Waltham, MA, USA) during the incubation. The lysis rate (OD min^-1^) was defined as the absolute value of the slope of the linear regression calculated on consecutive points on the steepest portion of the lysis curve.

### De-chaining assay

*S*. *gordonii* V288 and GP1485 were grown in TSB at 37°C. For each strain, 2-ml culture aliquots were collected at the same OD_590_ value multiple times during growth. The OD_590_ values selected were: 0.2, 0.8, 1.9, and ~1.7 (ON). At each collection point, culture aliquots were frozen at -80°C in 10% (v/v) glycerol. At the time of the assay, bacterial cells were defrosted, centrifuged at 3,500 × *g* at 4°C for 5 min, washed in 1× PBS, and resuspended in Tris 50 mM pH 7.0 at a cell concentration of ~ 2 × 10^8^ cells ml^-1^. The de-chaining activity of recLytB was tested by adding the enzyme to the GP1485 cell suspension at a final concentration of 0.2 mg ml^-1^. GP1485 and V288 cell suspensions without enzyme were used as controls. Samples were incubated at 37°C for 6 h. After incubation, a 10 μl aliquot of each bacterial suspension was spotted on a glass slide, stained with crystal violet, and observed by light microscopy with a Leica DM1000 microscope at 100× magnification. For each sample, at least three pictures of a representative field were captured with a Leica DFC 490 digital camera, and used to count the chain length. The chain length was expressed as the mean ± SEM of the number of bacterial cells forming a chain.

### Cell fractionation

Bacterial cell fractions of *S*. *gordonii* V288 were prepared as described [27]. Briefly, four different fractions were obtained: (i) cell wall, which contains cell wall fragments released following lysozyme and mutanolysin digestion; (ii) envelope, which represents membranes and cell wall-membrane fragments; (iii) clarified cytoplasm, (iv) culture supernatant. *S*. *gordonii* cultures were grown to early stationary phase and sedimented by centrifugation. Bacteria were washed once, resuspended in 0.1 ml of protoplasting buffer (100 mM Tris, pH 7.2; 30% (w/v) sucrose; 5 mM EDTA; 5 mM dithiothreitol) containing 1 mM PMSF, 1 mg ml^-1^ lysozyme (Sigma-Aldrich, St Louis, MO, USA), and 100 U ml^-1^ mutanolysin (Sigma-Aldrich, St Louis, MO, USA), and incubated at 37°C with gentle shaking for 1h. Protoplasts were centrifuged at 16,000 × *g* for 2 min. The recovered supernatant represented the cell wall fraction. Protoplasts were lysed by performing five washes in sterile distilled water. The supernatant collected in each step was pooled and ultra-centrifuged at 100,000 × *g* at 10°C for 30 min in a Beckmann Coulter Optima LE-80K ultracentrifuge. The pellet, representing the envelope fraction, was resuspended in 0.1 ml of 0.25 M Tris HCl pH 6.8,. The supernatant was precipitated with 20% (w/v) TCA by centrifugation at 13,000 × *g*, at 4°C, for 30 min. The recovered pellet was resuspended in 0.1 ml of 0.25 M Tris pH 6.8 to obtain the clarified cytoplasmic fraction. Culture supernatant was filtered (0.22 μm), loaded in an Amicon® Ultracel device (Millipore, Billerica, MA, USA) with 10 KDa membrane cut off and concentrated (53 ×) according to manufacturer instructions. Cell fractions (~1 × 10^10^ cell equivalents) were separated on a NuPAGE 4–12% Bis-Tris acrylamide gels and transferred on nitrocellulose membranes. The presence of LytB was detected with an anti-LytB mouse serum (1:1,000 dilution), and with an anti-mouse IgG serum (1:10,000 dilution) conjugated with alkaline phosphatase (Sigma-Aldrich, St Louis, MO, USA).

### Co-culture

*S*. *gordonii* V288 and GP1485 were inoculated from frozen stocks in 10 mL of TSB in sealed 16mm-diameter tubes and incubated at 37°C. Growth was monitored by a Spectronic 20D+ spectrophotometer. When bacterial cultures reached an OD_590_ value of 0.020, a volume of 5 mL of each culture was sampled, transferred in a new tube and co-cultured to early stationary phase (OD_590_ 1.080). Chain length was determined by directly spotting 10 μl of the co-culture on a glass slide. Equal volumes of V288 and GP1485 cultures were mixed and spotted to the glass slide, as controls. Bacteria were heat-fixed, stained with crystal violet and observed by using a light microscope at 100× magnification. For each sample, at least three pictures of a representative field were captured with a Leica DFC 490 digital camera and used to count the chain length. The chain length was expressed as the mean ± SEM of the number of bacterial cells forming a chain.

### *In-vitro* biofilm assay

Biofilm formation was analyzed in 6 well flat bottom polystyrene tissue culture plates (Sarstedt, Newton, NC, USA). Bacteria from frozen stocks (~ 4 × 10^8^ CFU ml^-1^) were inoculated in the wells containing 5ml of TSB. Plates were incubated at 37°C in a 5% CO_2_ enriched atmosphere. At 2, 4, 6, 8, 10 and 24 h. plates were analyzed for biofilm formation. Planktonic cells were removed and each well was washed 3 times with 5 ml of PBS in order to remove loosely attached bacteria. Plates were dried at 30°C for 15 min and stained with 0.5 ml of crystal violet for 15 min. Wells were washed with distilled water and dried for a second time. Biofilm was removed with 1 ml of absolute ethanol and quantified by measuring the absorbance at OD_590_.

### Statistical analysis

Two tailed Student’s t-test was used to analyze the data; statistical significance was defined as *P* < 0.05.

## Results

### Identification of LytB

**Fig 1:**
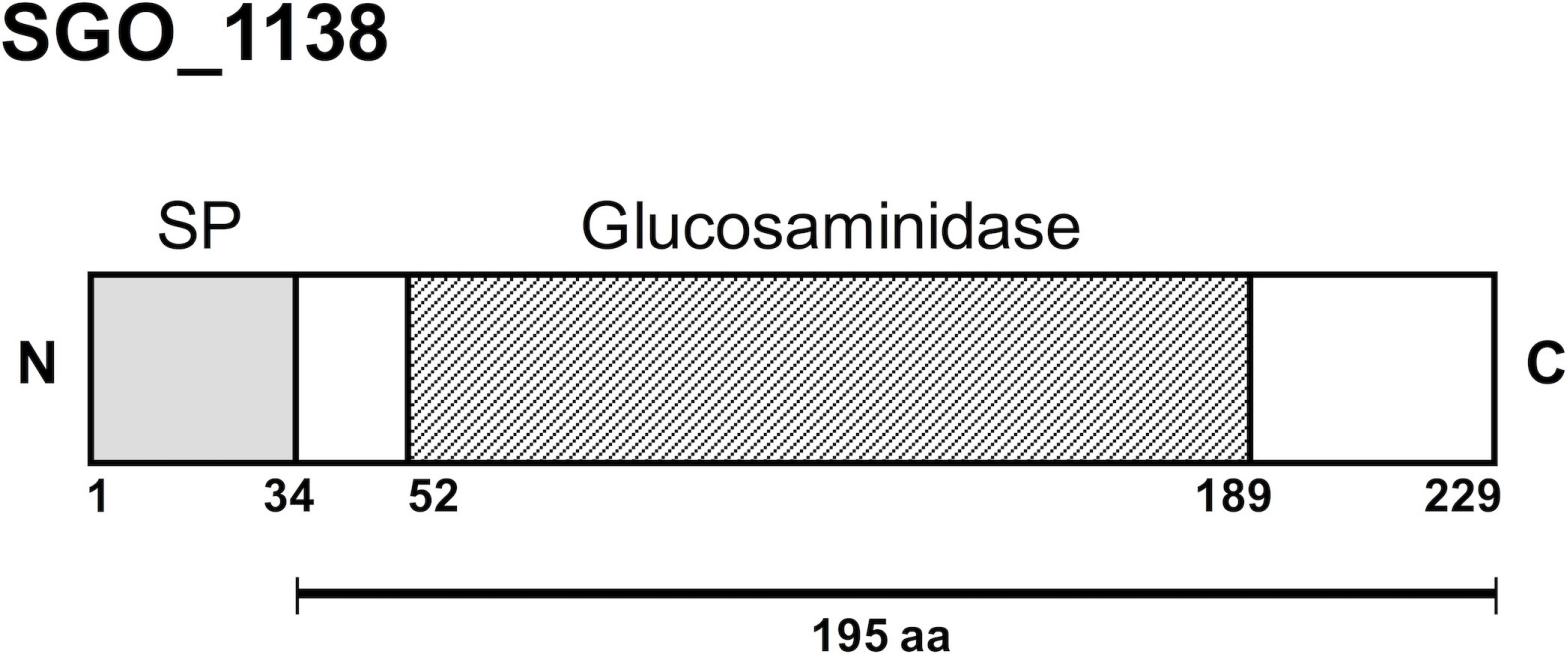
Domain organization of SGO_1138 of *S*. *gordonii* Challis (NCBI RefSeq: WP_012000547.1). Domains and amino acid positions were assigned with Pfam (http://pfam.sanger.ac.uk/) and drawn to scale. Numbers indicate the amino acid positions. SGO_1138 (229 amino acids) contains a 34 amino acids signal peptide (SP) and, a 137 amino acids glucosaminidase domain (Pfam PF01832). The 195 amino acid segment expressed in *E*. *coli* is indicated.

**Fig 2:**
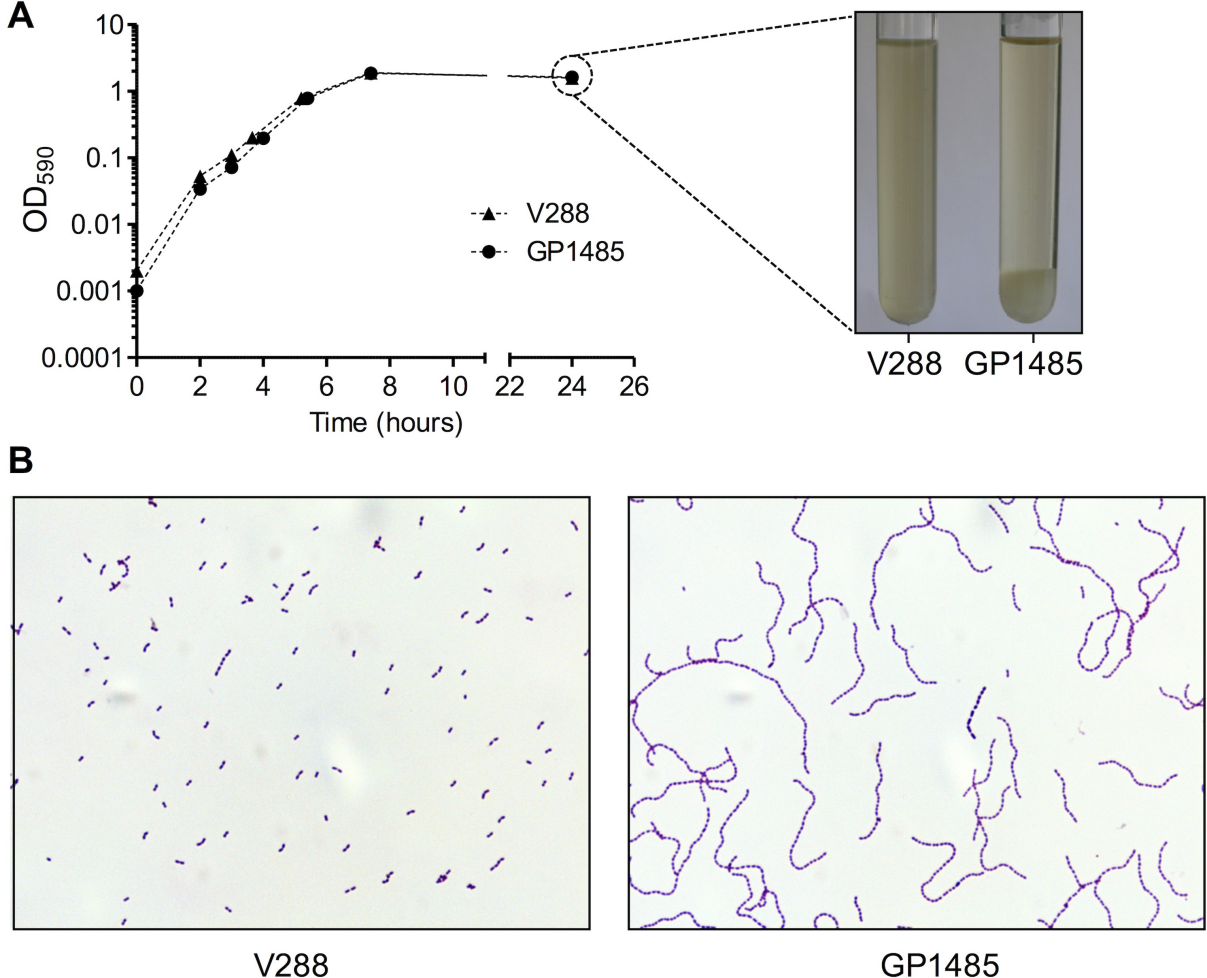
Growth curve and microscopic analysis of wild type and *lytB* mutant *S*. *gordonii*. (A) *S*. *gordonii* V288 and GP1485 growth curves. Strains were inoculated from frozen stocks in tubes containing 10 ml of TSB, and growth was monitored spectrophotometrically for 24 h. Parallel tubes were incubated statically and photographed after 24 h of incubation. (B) Long chain phenotype of *S*. *gordonii lytB* mutant GP1485. Early stationary phase *S*. *gordonii* cultures (OD_590_ 1.1) were spotted onto a glass slide, heat-fixed and stained with crystal violet. Samples were observed with a light microscope at 100× magnification. Pictures are representative of the whole bacterial population. Data from one representative experiment (of three independent experiments) are shown.

The *lytB* gene of *S*. *pneumoniae* has been previously shown to encode a putative endo-beta-N-acetylglucosaminidase that functions as a chain-dispersing enzyme [5]. The genome of *S*. *gordonii* does not encode a homolog of *S*. *pneumoniae lytB*. However, a search for glucosaminidase catalytic domains (Pfam PF01832) yielded *SGO_1138* (gene ID: 25052579) in the chromosome of *S*. *gordonii* Challis strain (NCBI RefSeq: NC_009785). *SGO_1138* is a 627 bp ORF that encodes for a 229-amino acids polypeptide characterized by a putative 34-amino acids signal peptide and a 137-amino acid glucosaminidase catalytic domain (Pfam PF01832) (Fig 1). To investigate whether *SGO_1138* was involved in daughter cell separation, we constructed a *SGO_1138* deletion mutant (GP1485) and characterized its growth phenotype. GP1485 showed a growth curve similar to wild type *S*. *gordonii* strain Challis V288 in liquid medium (Fig 2A). When the early stationary phase bacterial cultures were observed under a light microscope, we observed that the wild type strain grew in short chains and diplococci, whereas GP1485 formed long bacterial chains and aggregates that were dispersed by vortexing (Fig 2B). The GP1485 mutant phenotype resembles that observed with the *lytB* mutation of *S*. *pneumoniae*. Thus, *SGO_1138* was renamed *lytB*.

### LytB muralytic activity on *M*. *lysodeikticus* cells

**Fig 3:**
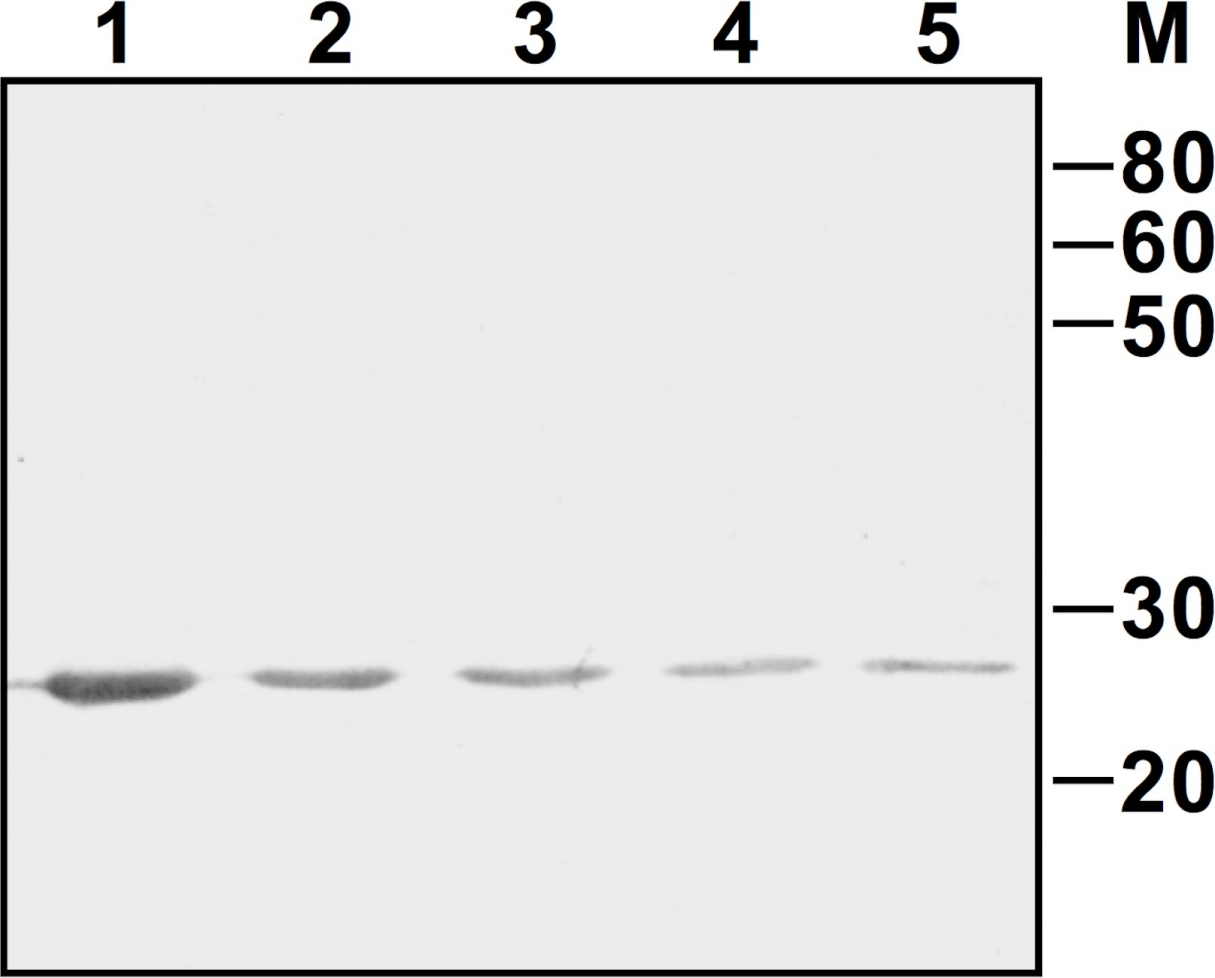
Western blot analysis of recLytB. Membrane was incubated with anti-His tag antibodies. Two-fold dilutions of purified recLytB ranging from 1 μg to 0.0625 μg are displayed (Lane 1–5). Numbers on the side indicate the molecular mass marker expressed in KDa.

**Fig 4:**
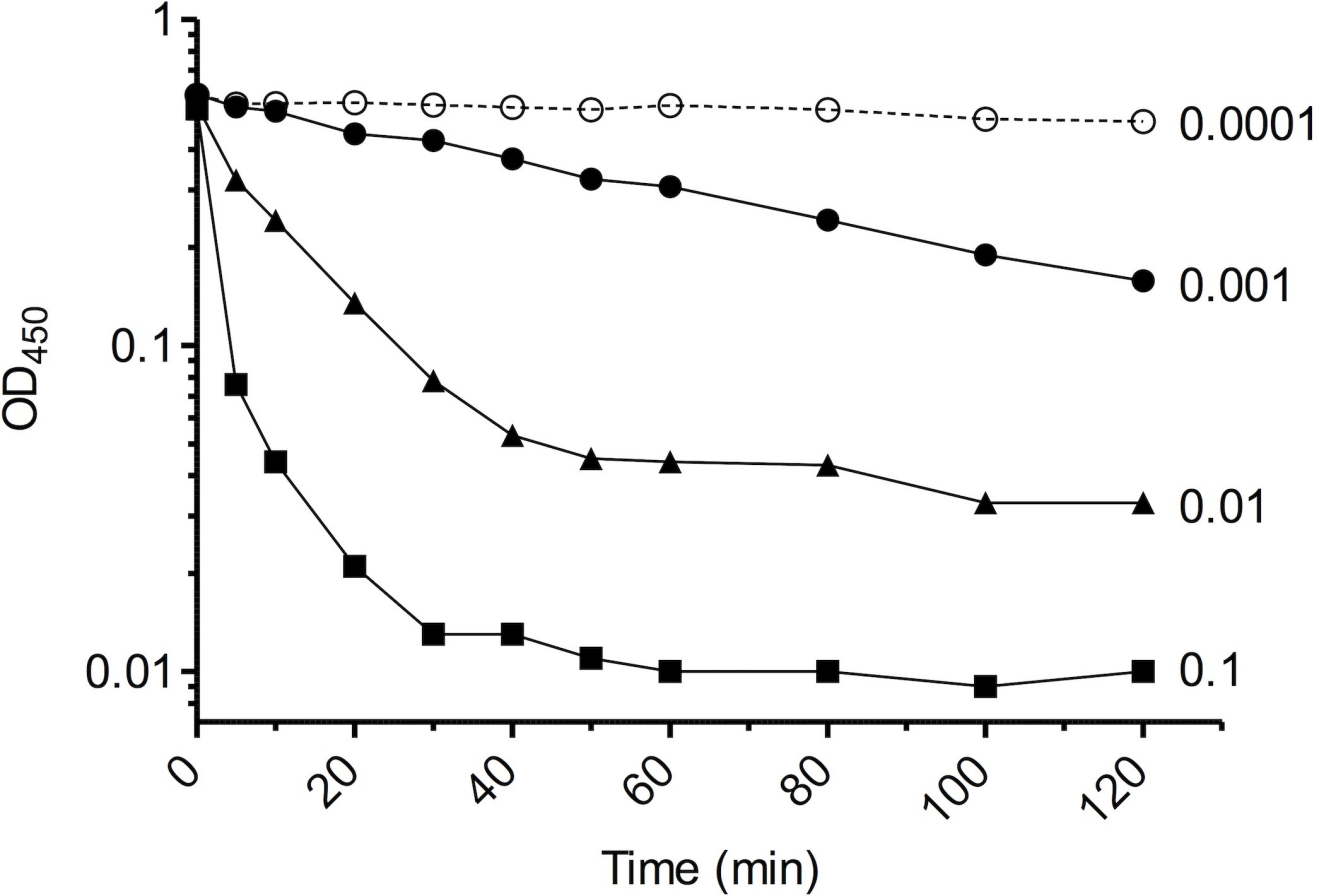
Muralytic activity of recLytB. Ten-fold serial dilutions of recLytB were added to a whole-cell suspension of *M*. *lysodeikticus*. Lytic activity of recLytB on *M*. *lysodeikticus* cells was monitored over time by measuring OD_450_ using a spectrophotometer. Data from one representative experiment (of three independent experiments) are shown.

The mature form of LytB was produced in *E*. *coli* as a fusion with a C-terminal His tag, and purified by affinity chromatography under native conditions. The expected molecular mass (25.1 KDa) of recLytB was confirmed by Western blot analysis (Fig 3). The lytic activity of recLytB was tested on a whole-cell suspension of *M*. *lysodeikticus* using ten-fold serial dilutions of the recombinant enzyme, ranging from 0.1 to 0.0001 mg ml^-1^ (Fig 4). When recLytB was added at a concentration of 0.1 mg ml^-1^, the lysis rate was 260-fold higher than that of the untreated control, and 98.1% of the bacterial population was lysed after 2 hours of incubation. The lytic activity was clearly dose-dependent and was still present at a concentration of 0.001 mg ml^-1^ (Table 1).

**Table 1:** Muralytic activity of recLytB.

LytB concentration (mg ml^-1^)	Lysis Rate		Lysis Outcome	
	OD min^-1^^*a*^	Fold increase^*b*^	Final OD^*c*^	% of lysis^*d*^
0.1	0.091	260	0.010	98.1
0.01	0.030	85.7	0.033	93,9
0.001	0.0039	11.1	0.158	73,2
0.0001	0.00058	1.7	0.486	17.1
None	0.00035	1	0.478	4.0

^*a*^Lytic rate was expressed as the absolute value of the slope of the linear regression calculated on consecutive points in the steepest portion of the lysis curve (Fig 4).

^*b*^Fold increase of lysis rate relative to the control with no enzyme.

^*c*^OD_450_ value measured after 2 h of incubation.

^*d*^Percentage of OD decreases after 2 h of incubation.

### Functional complementation of the Δ*lytB* mutant by recLytB

**Fig 5:**
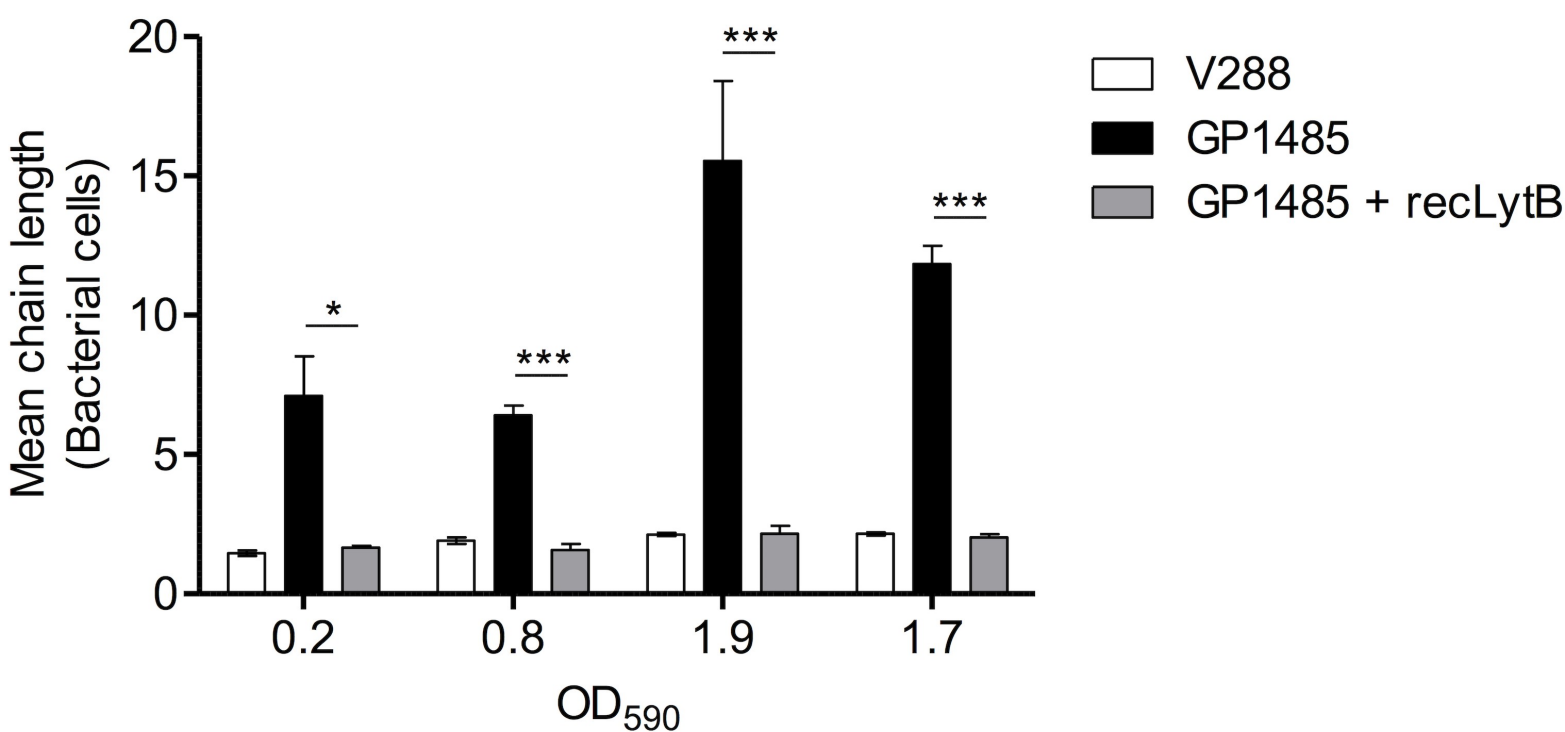
De-chaining activity of recLytB. Aliquots from cultures of *S*. *gordonii* V288 and Δ*lytB* mutant GP1485 were collected at the following OD_590_ values: 0.2, 0.8, 1.9 and ~1.7 (ON) (since the growth curves of the two strains were essentially indistinguishable (see Fig 1), same OD_590_ values corresponded to the same time points). Cells were washed, resuspended in buffer Tris 50 mM pH 7.0, and incubated with or without recLytB at 37°C. V288 without recLytB (white bar), GP1485 without recLytB (black bar), GP1485 with 0.2 μg μl^-1^ of recLytB (gray bar). After 6 hours of incubation, bacteria were observed by light microscopy to count the number of chain-forming bacteria. Data are expressed as the mean ± SEM of the number of cells forming a chain (mean chain length) in at least three microscopy fields. The asterisks indicate *P < 0.05 and ***P < 0.001. Data from one representative experiment (of two independent experiments) are shown.

The capacity of recLytB to complement the chain-dispersing function, which is lacking in the *lytB* knock-out mutant GP1485, was tested by adding purified LytB to the mutant. Aliquots from cultures of wild type and mutant strains were collected at the same OD_590_ values during growth. At each OD_590_ tested, addition of LytB caused a significant reduction (3- to 7- fold) of the chain length of GP1485 (*P* < 0.05) (Fig 5). The resulting phenotype, which was characterized by short chains, diplococci and single bacterial cells, was indistinguishable from that of wild type cells (Fig 5).

### Sub-cellular localization of LytB and complementation of the Δ*lytB* mutant phenotype in co-culture experiments

**Fig 6:**
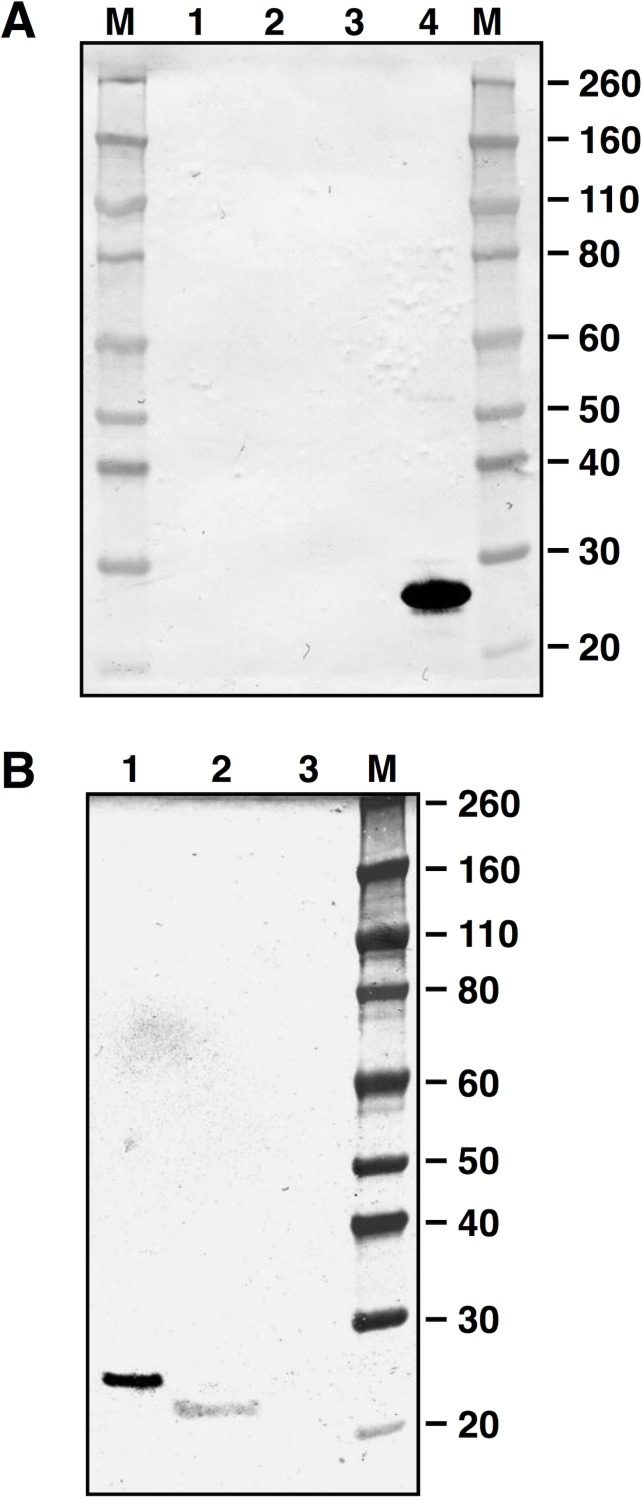
LytB sub-cellular localization. Membranes were incubated with an anti-LytB specific serum raised in mice. Numbers on the sides indicate the molecular mass marker (M) expressed in KDa. (A) Lane 1, cell wall; lane 2 envelope; lane 3 cytoplasm; lane 4, 1 μg of recLytB. Fractions loaded in each well corresponds to 1 × 10^10^ cell equivalents. (B) Lane 1, 0.2 μg of recLytB; lane 2, culture supernatant of V288; lane 3, culture supernatant of Δ*lytB* mutant GP1485.

**Fig 7:**
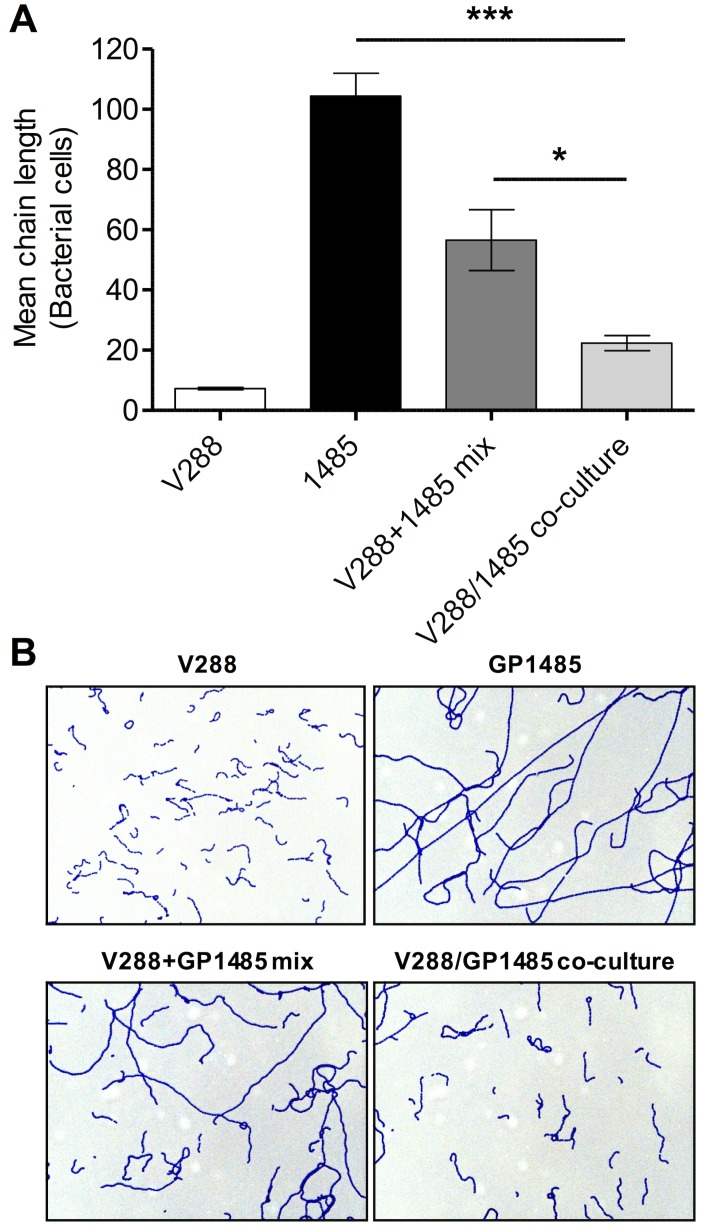
V288 and Δ*lytB* mutant co-culture. (A) Co-culture assay. *S*. *gordonii* wild type strain V288 (white bar) and *lytB* mutant GP1485 (black bar) were grown separately or in co-culture (light grey bar) and spotted directly on a glass slide for chain enumeration. An equal culture volume of V288 and GP1485 was mixed and spotted on a glass slide as control (dark grey bar). Data were expressed as the mean ± SEM of the number of cells forming a chain (mean chain length) considering at least three microscopy fields. The asterisks indicate *P < 0.05 and ***P < 0.001. Data from one representative experiment (of two independent experiments) are shown. (B) Representative microscopy pictures of the conditions described in panel A.

We next determined the sub-cellular localization of LytB by assaying bacterial cell fractions of *S*. *gordonii* V288 and Δ*lytB* mutant GP1485 for the presence of LytB by Western blot analysis. LytB was detected only in wild type culture supernatants (Fig 6A and 6B). Since LytB was found in culture supernatants, we reasoned that co-culturing the wild type strain with **Δ***lytB* mutant strain might complement the absence of LytB in GP1485. After 8 hours of co-culture, the mean chain length of mutant GP1485 was significantly decreased (~ 5-fold) relative to GP1485 culture alone (Fig 7A and 7B). Mixing the two cultures immediately prior to microscopy analysis only resulted in halving the chain length, as expected (Fig 7A and 7B), indicating that the effect of co-culturing on chain length results from enzymatic activity rather than the mere presence of both wild type and mutant cells in the mix.

### The Δ*lytB* mutant is impaired in biofilm formation

**Fig 8:**
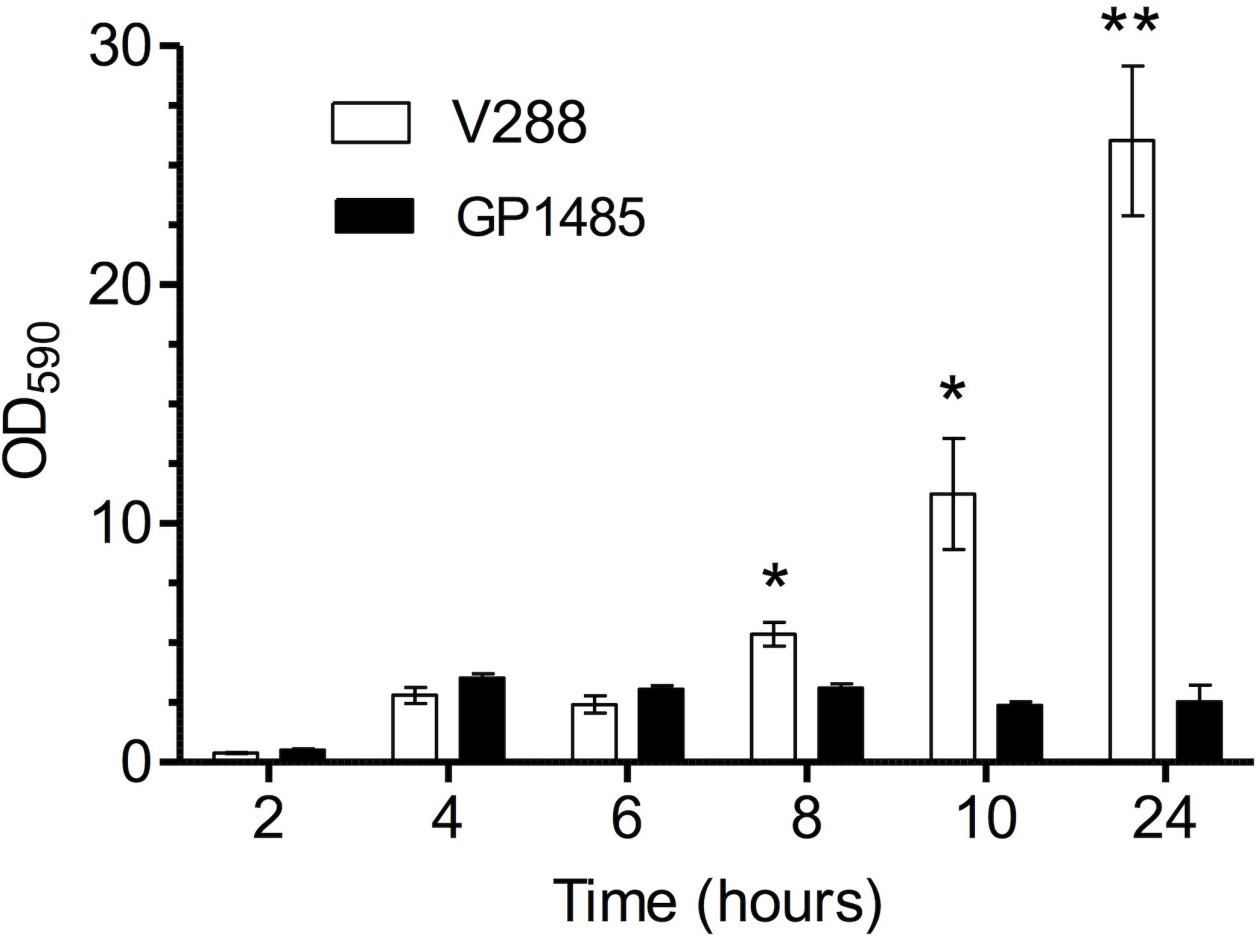
*In-vitro* biofilm kinetic assay. Biofilm formation was assayed in 6 well polystyrene tissue culture plates. The wild type V288 and Δ*lytB* mutant GP1485 strains were inoculated from frozen stocks and incubated at 37°C in a CO_2_ enriched atmosphere. At 2, 4, 6, 8, 10 and 24 hours of incubation, plates were washed and stained with crystal violet. Biofilm was removed with ethanol and quantified at 590 nm wavelength. Data represent the mean ± SEM of two experiments performed in triplicate. The asterisks indicate *P < 0.05.

To investigate whether the **Δ***lytB* mutant was affected in biofilm formation, the isogenic pair (mutant GP1485 and wild type V288) was tested in an *in-vitro* kinetic assay of biofilm formation, with bacteria growing in liquid medium in six-well tissue culture plates. During the exponential phase of growth (2 to 6 hours after the inoculum), wild type and mutant produced the same amount of biofilm, while in late exponential phase (8 hours), early stationary phase (10 hours), and late stationary phase (24 hours), wild type V288 produced from 2- to 10-fold higher amount of biofilm relative to the Δ*lytB* mutant (Fig 8).

## Discussion

Numerous observations in the literature have clearly established the essential role of PGHs during bacterial cell separation. Mutants deficient in cell-separating PGHs can replicate but grow in long chains of cells [5–11]. Here we report that the genome of the oral commensal *S*. *gordonii* carries *lytB*, a gene coding for a putative glucosaminidase. Even though *lytB* mutant grew at the same rate of the wild type strain, it was impaired in cell separation and showed a long-chain phenotype. Addition of recombinant LytB to a *lytB* deficient mutant functionally complemented the mutant phenotype, demonstrating that *S*. *gordonii* LytB functions as a cell-separating enzyme. Since the de-chaining activity of *S*. *gordonii* LytB does not lead to cell lysis (not shown), future experimentation will test the putative polar localization of this enzyme.

Our work shows that LytB of *S*. *gordonii* has unique properties among PGH. LytB is presumably a secreted protein, since it is found only in the bacterial culture supernatant and carries a putative secretion signal peptide. Moreover, LytB carries no recognizable conserved peptidoglycan binding domains. The reduction of the mean chain length in the *lytB* mutant observed when wild type and mutant where co-cultured is consistent with the proposed secreted nature of this protein. In contrast, PGHs involved in cell separation are typically localized on the peptidoglycan by single or multiple binding domains. For example, a single LysM domain targets Cse of *S*. *thermophilus* [6] and AcmA of *L*. *lactis* [28] directly to the peptidoglycan, while *S*. *pneumoniae* LytB binds to choline residues on teichoic acids by repeated choline binding domains (ChBD) [29]. Among the PGHs lacking specific cell-wall-binding domains, *L*. *plantarum* Acm1 does not alter chain length [11], while *S*. *thermophilus* Mur1, *L*. *lactis* AcmC, and *Leuconostoc citreum* Mur have not been demonstrated to be cell-separating enzyme due to the lack of mutational or gene inactivation studies [30–32]. Based on these considerations, LytB of *S*. *gordonii* constitutes a novel example of an extracellular cell-separating enzyme.

*S*. *gordonii* is a close relative of the human pathogen *S*. *pneumoniae* [16]. A *S*. *pneumoniae lytB* mutant has reduced adhesion properties when tested in biofilm assays *in vitro* [33] and *in vivo* [34]. In our experimental biofilm model, *S*. *gordonii lytB* mutant resulted impaired in late stages of biofilm formation compared to the wild type. Since the maximum chain length of the mutant was measured during the late stages of growth, we hypothesize that the reduced adhesion of the mutant is a consequence of the shearing forces to which the long chains of *lytB* mutant are subjected. In the oral cavity, mouth fluid flow, tongue movements, and other physiologic movements of the mouth represent shear forces to which bacteria are exposed during the initial adhesion to the tooth [35, 36]. Thus, our results indicate that bacterial chain length can be a contributing factor to the success of *S*. *gordonii* adherence, pointing to the possibility that arrangement of streptococci in long chains can affect the initial colonization of tooth surfaces and the interaction with other bacterial species of dental biofilms.
